# Distinct temporal trends in breast cancer incidence from 1997 to 2016 by molecular subtypes: a population-based study of Scottish cancer registry data

**DOI:** 10.1038/s41416-020-0938-z

**Published:** 2020-06-19

**Authors:** Ines Mesa-Eguiagaray, Sarah H. Wild, Philip S. Rosenberg, Sheila M. Bird, David H. Brewster, Peter S. Hall, David A. Cameron, David Morrison, Jonine D. Figueroa

**Affiliations:** 1 Usher Institute, College of Medicine and Veterinary Medicine, University of Edinburgh, Edinburgh, UK; 2 Division of Cancer Epidemiology and Genetics, National Cancer Institute, Bethesda, MD USA; 3 Cambridge University’s MRC Biostatistics Unit, Cambridge, CB2 0SR UK; 4 Cancer Research UK Edinburgh Centre, Institute of Genetics and Molecular Medicine, University of Edinburgh, Edinburgh, UK; 5 NHS National Services and University of Glasgow, Glasgow, UK

**Keywords:** Cancer epidemiology, Tumour biomarkers

## Abstract

**Background:**

We describe temporal trends in breast cancer incidence by molecular subtypes in Scotland because public health prevention programmes, diagnostic and therapeutic services are shaped by differences in tumour biology.

**Methods:**

Population-based cancer registry data on 72,217 women diagnosed with incident primary breast cancer from 1997 to 2016 were analysed. Age-standardised rates (ASR) and age-specific incidence were estimated by tumour subtype after imputing the 8% of missing oestrogen receptor (ER) status. Joinpoint regression and age–period–cohort models were used to assess whether significant differences were observed in incidence trends by ER status.

**Results:**

Overall, ER-positive tumour incidence increased by 0.4%/year (95% confidence interval (CI): −0.1, 1.0). Among routinely screened women aged 50–69 years, we observed an increase in ASR from 1997 to 2011 (1.6%/year, 95% CI: 1.2–2.1). ER-negative tumour incidence decreased among all ages by 2.5%/year (95% CI: −3.9 to −1.1%) over the study period. Compared with the 1941–1959 birth cohort, women born in 1912–1940 had lower incidence rate ratios (IRR) for ER+ tumours and women born in 1960–1986 had lower IRR for ER− tumours.

**Conclusions:**

Future incidence and survival reporting should be monitored by molecular subtypes to inform clinical planning and cancer control programmes.

## Background

Breast cancer incidence is rising and it is the most common cancer among women worldwide.^1^ Breast cancer is not a single disease, but comprises multiple subtypes, with oestrogen receptor (ER) expression, a key marker of prognostic and aetiologic significance.^2^ ER+ tumours, which are amenable to targeted anti-oestrogenic therapies, such as tamoxifen and aromatase inhibitors, are the most common type of breast cancers accounting for 65–75% of breast cancer cases in high-income populations.^3^ Progesterone receptor (PR) is also a commonly tested marker of hormone responsiveness that is highly correlated with ER. Tumour overexpression of the human epidermal growth factor receptor 2 (HER2) was identified over two decades ago. The discovery of HER2 laid the foundation for biological therapies, which were shown to be clinically effective in treating tumours expressing this marker. HER2-targeted therapies have been widely available in the United Kingdom since 2006.^4^ ER− tumours are rarer, have an earlier age of onset and worse prognosis than ER+ tumours, in part because fewer targeted treatments are available than for ER+ tumours. In addition to prognostic differences, epidemiologic studies have shown aetiologic differences by tumour subtypes.^5,6^

There are relatively few population cancer registries that collect ER, PR and HER2 data, the key distinguishing markers for molecular subtypes of breast cancer. Recent analyses support divergent incidence trends by ER status in the United States, Denmark and Ireland, with ER+ breast cancer incidence increasing and ER− breast cancer incidence decreasing.^7–9^ Data on a combination of subtypes using ER, PR and HER2 are even more limited, with few reports from the United Kingdom.^10–12^ ER, PR and HER2 molecular markers are used often as surrogates for the intrinsic subtypes of breast cancer defined by mRNA expression profiling^13^ because, unlike genetic profiling subtypes, the molecular markers have been measured routinely in recent years. In the age of precision medicine, quantifying and monitoring cancer incidence by molecular subtypes are important in optimising public health prevention programmes, the allocation of resources and availability of screening, diagnostic and therapeutic services and for improving outcomes.^14^ An important issue in assessing trends by ER status is the need to account for missing data, as completeness of marker data has improved over time, but imputation methods can be applied to address this limitation.^7–9,15^

Within Scotland’s renowned, high-quality routine electronic health records, the Scottish cancer registry is an excellent resource to investigate temporal trends in cancer incidence. Data collection began for ER in 1997 and PR and HER2 in 2009, and so provides data almost a decade earlier than other UK national registries. While monitoring of breast cancer incidence in the United Kingdom is standard,^16,17^ these data have not been presented by molecular subtypes, despite substantial evidence that heterogeneity exists by ER status.^6,18–20^

Here we report on breast cancer incidence trends in Scotland by ER and ER/HER2 combinations using several statistical methods: (1) age-standardised and age-specific incidence rates, which are typically used to report cancer statistics,^21^ (2) joinpoint regression models to determine whether significant changes occurred during 1997–2016, and the speed at which they have occurred^22^ and (3) age–period–cohort (APC) models^23–25^ based on generalised linear model theory to enable description of age, period and birth cohort effects to provide possible clues to potential underlying factors contributing to incidence trends, and thereby inform public health and NHS programmes.

## Methods

### Data and cohort definition

All primary invasive breast cancers (defined on the basis of the International Classification of Diseases, 10th revision code of C50) diagnosed in women aged 20+ years, between 1997 and 2016, were ascertained from the Scottish cancer registry held by Information Services Division (ISD) of NHS National Services Scotland. The Scottish cancer registry achieves 98% breast cancer case ascertainment and is over 99% complete.^26^ Breast cancer incidence after a previous cancer is considered a different aetiology (i.e. possible different risk factors such as radiation exposure amongst others) and, while an interesting topic, was not the major interest of this analysis. Supplementary Fig. 1 describes how the final study population was derived: notably, by exclusion of men and women who had a prior non-breast primary tumour. ER status was based on Allred scoring.^27^ Women with primary breast cancer are the basis for analysis, each characterised by her worst-prognosis tumour. In our study population, 3653 women (5% of the study population) had multiple invasive breast cancers denoted in the cancer registry. The first primary invasive breast cancer was chosen if the time between diagnoses was >6 months, whereas for those with more than 1 diagnosis <6 months apart (*n* = 2094), the more advanced invasive cancer was selected. Of those 2094, 1837 (88%) had the same ER status, 154 (0.07%) had different ER status and the rest had one or more of the records with missing ER status. We therefore prioritised the record with less missing data. Given that only 0.07% cases lacked agreement in ER status, this prioritisation had negligible impact on the results; using tumours (not individuals) as the numerator, which is typically done in regular cancer reporting, overestimates the incidence rates of breast cancer, and we used one tumour per person to minimise bias in time trends. Permission for use of the data was obtained from the Public Benefit and Privacy Panel (PBPP) of NHS Scotland (reference number 1718-0057), and analyses were conducted in the Scottish National Safe Haven.^28^

Additional demographic and tumour data obtained were age at diagnosis, NHS Scotland regions (North, South East and West), tumour grade (grade I—well differentiated to III—poorly differentiated), tumour size (less than 10 mm, 10–20 mm and more than 20 mm), nodal involvement (yes or no), screen-detected tumour (yes or no) and the status of molecular markers ER, PR and HER2 (positive, negative or unknown). ER and PR status are measured using immunohistochemistry (IHC), and HER2 status was assessed using a combination of IHC with fluorescent in situ hybridisation for equivocal (2+) cases. Previous studies have noted that assessment of ER status reliability is high with an error rate below 5%.^29^ ER/HER2 combinations were used as surrogates for the four intrinsic subtypes of breast cancer, the gold standard for which uses mRNA expression profiling. ER+/HER2− was used as a surrogate for Luminal A tumours, ER+/HER2+ for Luminal B, ER−/HER2+ for HER2-enriched tumours and ER−HER2− for triple-negative tumours. The high quality of these data has been previously described.^30^

### Statistical methods

Missing ER and ER/HER2 status were imputed conditioned on age and year of diagnosis, with the assumption that data were missing at random, using a validated method.^7–9^ Age-standardised incidence rates (ASR) per 100,000 women were calculated using the direct method, the European standard population (2013) and mid-year estimates of the Scottish population for each age and year.^31^ Age-specific incidence rates were calculated for 5-year age groups (20–24 to 90+) and individual calendar years using two approaches: with the number of tumours as the numerator for consistency with routine reporting, and with one tumour per woman as the numerator for all other analyses. ASRs were calculated for all age groups combined and for three separate age groups, with the middle group defined on the basis of eligibility for routine breast screening in Scotland (20–49 years, 50–69 years and 70 years or older), and for each ER status and ER/HER2 combinations.

Joinpoint regression models were used to describe breast cancer incidence rates overall, by ER status and ER/HER2 combinations for all women in the cohort and for three age groups (20–49, 50–69 and 70+ years). Joinpoint models describe if changes in incidence trends occur and identify the time points at which a change is observed (referred to as joinpoints). The permutation test method, as described by Kim et al.,^22^ was used iteratively: it starts by testing the null hypothesis of a simple model with zero joinpoints against the alternative hypothesis of a more complex model with the maximum number of joinpoints previously specified (3 joinpoints for this study). The procedure continues until all possible numbers of joinpoints have been tested. A total of 4,499 permutations are performed, and the p-value test is adjusted for multiple testing using the Bonferroni correction.^32^ In the final model, the estimated annual percentage change (EAPC) for each of the periods identified is calculated. The average annual percent change (AAPC) is also reported as a measure of the overall trend from 1997 to 2016. Joinpoint regression software is a free open- access software that can be downloaded at https://surveillance.cancer.gov/joinpoint/.^33^

APC models were fitted for age-standardised incidence of ER+ and ER− tumours. The APC model provides a unique set of best-fitting log_10_ incidence rates obtained by maximum likelihood estimators for period, age and cohort, which have been shown to provide similar rates to ASR, but allow investigation of differences by birth cohorts—with the middle cohort as referent—which are not investigated in ASR or joinpoint regression analysis. As a consequence of small numbers in some strata, we restricted these models to women aged 30–85 years and used 28 2-year age groups (from 30–31 to 84–85) and 10 2-year periods (from 1997–1998 to 2015–2016) of calendar year of diagnosis, which covered birth cohorts from 1912 to 1986. The net drift, similar to the EAPC and AAPC estimates, is reported with 95% confidence intervals (CI). Local drifts were also estimated and describe the annual percentage change for each age-specific rate over time.^34^ In addition, period and cohort rate ratios are also presented to compare the age-specific rates in each period or cohort with the reference points in the middle of the study period and birth cohort (2006 for period and 1949 for cohort). Together with cohort rate ratios (CRR), a combination test of significance for the complete cohort deviations is reported. This new combination test aims to determine if there is an association of the observed rates with the birth cohorts above the linear influences represented by the net drift. The test provides a more robust method than the traditional Wald test while correcting for multiple testing. With the exception of joinpoint regression, all analyses were carried out using R.^35^

## Results

### Characteristics of the cohort by ER status

Between 1997 and 2016, 72,217 women of 20 years of age or older were diagnosed with at least one invasive breast cancer in Scotland (Table 1). Seventy-six percent of these tumours were ER+, 16% were ER− and 8% had unknown ER status. However, the percentage of missing ER status decreased over time from 20% in 1997 to 2% in 2016. Proportions with unknown ER status differed by region and age: higher in the West compared with the North and Southeast of Scotland and in women aged 70 years or older compared with women younger than 70 years (14% missing vs. 5%). Almost half of breast cancers were diagnosed among women of 50–69 years of age, similar to the range for eligibility for routine breast cancer screening (50–70 years) since 2003.

**Table 1:** Descriptive characteristics by ER status for all women with an invasive breast cancer diagnosed between 1997 and 2016 in Scotland.

Characteristics	ER−		ER+		ER unknown	
	*n*	%	*n*	%	*n*	%
	11,726	[16]	55,144	[76]	5347	[8]
Age at diagnosis						
<50 years	3196	(27)	10,550	(19)	695	(13)
50–69 years	5668	(48)	28,441	(52)	1580	(30)
70 years or older	2862	(24)	16,153	(29)	3072	(57)
Grade						
I—well differentiated	195	(2)	8288	(15)	232	(4)
II—moderately differentiated	1714	(15)	25,734	(47)	602	(11)
III—-poorly differentiated	8308	(71)	14,586	(26)	642	(12)
Unknown	1509	(13)	6536	(12)	3871	(72)
Nodal status						
Uninvolved/negative	6194	(53)	29,400	(53)	869	(16)
Involved/positive	4110	(35)	17,369	(31)	415	(8)
Unknown	1422	(12)	8375	(15)	4063	(76)
Tumour size						
Less than 10 mm	1017	(9)	6470	(12)	202	(4)
10–20 mm	3428	(29)	20,449	(37)	478	(9)
More than 20 mm	4960	(42)	18,168	(33)	512	(10)
Unknown	2321	(20)	10,057	(18)	4155	(78)
PR status^a^						
Negative	3803	(79)	3036	(12)	<10	(<1)
Positive	226	(5)	15,869	(62)	<10	(<1)
Unknown	764	(16)	6489	(26)	901	(99)
HER2 status^a^						
Negative	2761	(66)	18,709	(84)	36	(5)
Positive	1210	(29)	2553	(11)	10	(1)
Unknown	184	(4)	1129	(5)	725	(94)

Brackets [] indicate row percentages and parentheses () indicate column percentages for that category.

^a^Denotes markers that were recorded from 2009 to 2016, and the number of cases for those years = 31,099. Differences by known ER status for all characteristics were significantly different with *χ*^2^ *p* < 0.001.

Tumour characteristics differed by ER status, with ER– tumours having characteristics associated with more advanced/aggressive disease. ER− tumours had higher grade, were larger and more likely to have positive lymph node status. The patterns of other molecular markers also differed by ER status, with ER– tumours more likely to be PR− and HER2+ than ER+ tumours. In contrast, ER+ tumours were more likely to be PR+ and HER2− than ER− tumours.

**Fig. 1:**
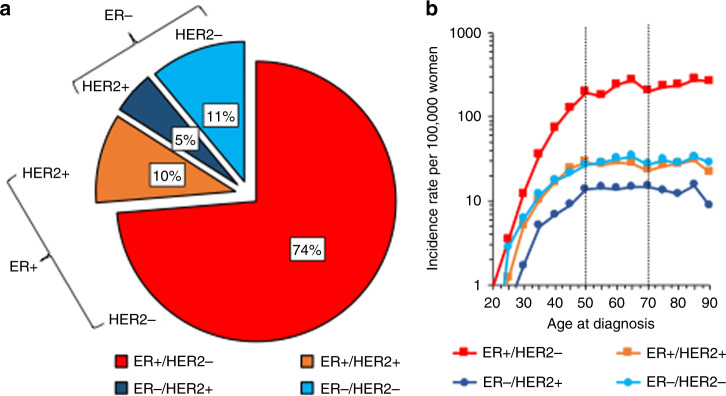
Distribution of the breast cancer subtypes by ER/HER2 status and their age-specific incidence in Scotland for 2009–2016 (*N* = 31,099). **a** Shows a pie chart and **b** shows age-specific incidence on the log scale by subtype. **b** Data are for 31,099 breast cancer cases with ER/HER2 missing status imputed for analysis. Dotted lines in the graph denote ages 50–70 years, the age group invited for screening in Scotland every 3 years.

The combinations of ER/HER2 status after imputing for missing ER and HER2 status are shown in Fig. 1a. Most tumours were ER+/HER2−, with ER−/HER2+ tumours being the least common combination. ER−/HER2− tumours, the most aggressive subtype, were the second most common at 11%. Cross-sectional age-specific curves for the ER/HER2 combinations (Fig. 1b) show incidence of all subtypes increasing rapidly with age, until the approximate age of menopause, age 50 years; thereafter, the increase continued more gradually up to 70 years for ER+/HER2− tumours, but there was no further increase for ER− tumours or ER+/HER2+ tumours.

#### Age-standardised incidence rates with EAPCs from joinpoint regression

Age-standardised incidence of ER+ tumours increased from 98 per 100,000 women in 1997 to 113 per 100,000 women in 2016 (Table 2, Supplementary Fig. 2), with an average annual percentage change (AAPC) of 0.4% (95% CI: −0.1 to 1%). Incidence was higher for ER+/HER2− tumours than for the rest of the subtypes, similar to that of ER+ tumours, with increases observed up to 2011. Estimates from the join-point analysis (Table 2) show that the increase in incidence of ER+ tumours was reasonably constant (1.2% increase annually, 95% CI: 0.8–1.5%) from 1997 till around 2012, after which incidence decreased by ~2.2% annually (95% CI: −4.7 to 0.4%). By contrast, ER− tumour incidence decreased over the study period by approximately 2.5% per year (95% CI: −3.9 to –1.1%), but showed a slow rate of decline of 0.7%/year (95% CI: −1.5, 0.0) from 2000 to 2016. ER−/HER2− tumour incidence increased by 3.2% (95% CI: 0.3–6.1%) from 2011 to 2016 (Supplemental Table 1 Supplementary Fig. 3), although the latter finding was based on relatively small numbers.

**Table 2:** Joinpoint regression analysis stratified by age groups and ER status from 1997 to 2016.

ER status	Age groups	Rate in 1997 per 100,000 women	Rate in 2016 per 100,000 women	Change in rate from 1997 to 2016 per 100,000 women (%)	Average annual percentage change (95% CI)	*N* for complete case analysis	*N* for estimated counts corrected for missing ER status	Years before joinpoint	EAPC (95% CI) for the period before joinpoint	Years after joinpoint	EAPC (95% CI) for the period after joinpoint
Positive	20–49	41.9	52.1	10.2 (20%)	1.1% (0.7, 1.5)	10,550	11,083	*No significant change point identified from 1997 to 2016*			
	50–69	192.3	237.4	45.1 (19%)	0.7% (0.2, 1.3)	28,441	29,758	1997–2011	1.6% (1.2, 2.1)	2011–2016	−1.8 (−3.7, 0.1)
	70+	235.9	234.5	−1.4 (−0.6%)	0.1% (−0.3, 0.5)	16,153	18,763	*No significant change point identified from 1997 to 2016*			
	All ages	97.7	112.8	15.1 (13%)	0.4% (−0.1, 1.0)	55,144	59,604	1997–2012	1.2% (0.8, 1.5)	2012–2016	−2.2 (−4.7, 0.4)
Negative	20–49	23.8	15.2	−8.6 (−36%)	–2.2% (−3.9, −0.6)	3196	3358	1997–2001	−10.0% (−17.0, −3.0)	2001–2016	0% (−1.1, 1.2)
	50–69	64.1	45.5	−18.6 (−29%)	−1.6% (−2.5, −0.8)	5668	5931	*No significant change point identified from 1997 to 2016*			
	70+	71.8	41.2	−30.6 (-–43%)	−2.4% (−4.2, −0.7)	2862	3324	1997–2003	−7% (−11.0, −2.0)	2003–2016	−0.3% (−1.9, 1.5)
	All ages	35.5	23.1	−12.4 (−35%)	−2.5% (−3.9, −1.1)	11,726	12,613	1997–2000	−11% (−19.0, −3.0)	2000–2016	−0.7% (−1.5, 0)

*EAPC* estimated annual percentage change, *AAPC* estimated average annual percentage change.

Joinpoint regression was performed using the estimated counts corrected for missing ER status, and analysis corrects for multiple testing using Bonferroni correction (see ‘Methods' section).

**Fig. 2:**
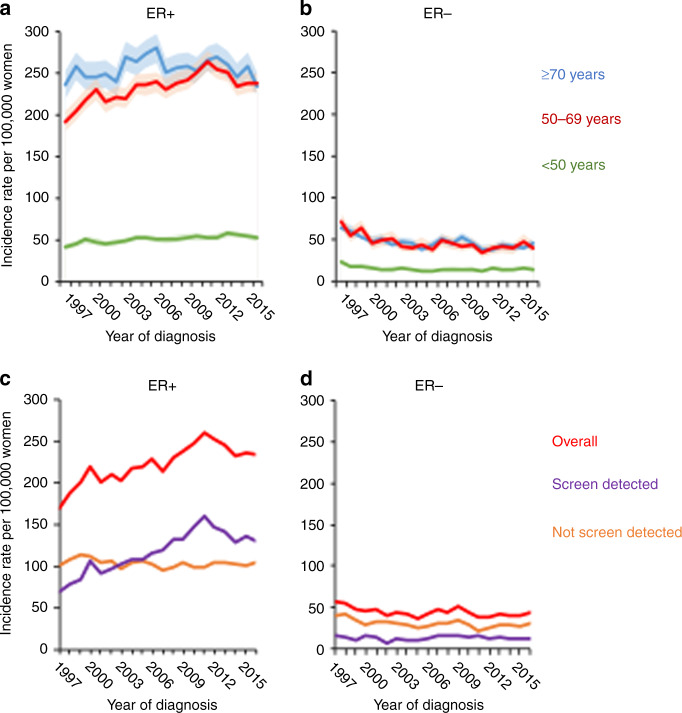
Age-specific trends in breast cancer incidence stratified by age groups, screen detection and ER status in Scotland for 1997–2016. ER-positive (**a**) and ER-negative (**b**) age-specific trends for age groups 20–49 (green), 50–69 (red) and 70 years old (blue). Shaded areas surrounding lines indicate 95% CI of rates. Panels **c**, **d** are restricted to women aged 50–69 years, and incidence rates overall (red), for screen-detected (purple) and not screen-detected (orange) are shown for ER+ (**c**) and ER− tumours (**d**).

Women 50–69 years of age had the highest increases in ER+ incidence at a similar period as noted overall (Table 2, Fig. 2a), followed by women aged 20–49 years where ER+ tumour incidence increased by 1.1% annually. For women of 70 years or older rates were stable. The decreases observed in ER− tumours were consistent across the three age groups (Fig. 2b). Differences in time trends in incidence rates were also observed between ER+ and ER− tumours, depending on whether the tumour was screen-detected or not. Among women aged 50–69 years with available ER and screening data, 53% of all ER+ tumours were screen-detected compared with 30% of ER− tumours. Further, among women aged 50–69 years with ER+ tumours, the incidence of non-screen-detected tumours was higher in earlier period years of diagnosis (1997–2003) than for screen-detected tumours. ER+ screen-detected tumours mimicked the incidence pattern observed for all ER+ tumours, with consistent increases in incidence until 2011, whereas non-screen-detected ER+ tumours remained constant (Fig. 2c). In women aged 50–69 years, the incidence of ER− tumours that were not screen-detected declined over time, whereas screen-detected ER− tumour incidence remained constant (Fig. 2d).

#### Age–period–cohort models

The results from APC models were consistent with those observed from joinpoint regression, with net drifts suggesting increases in the overall incidence of ER+ tumours by 0.8% per year (95% CI: 0.6–1.0%/year) from 1997 to 2016, and ER− tumour incidence decreasing by −1.4% (95% CI: −1.8 to −1.1%/year). After adjusting for period and cohort effects, local drifts showed that the highest increase in incidence of ER+ tumours was observed in women around 70 years of age (2% per year, 95% CI: 1.6–2.4%) (Supplementary Fig. 4a). The greatest drop in incidence of ER− tumours was observed in women of screening age 50–69 years (Supplementary Fig. 4b).

**Fig. 3:**
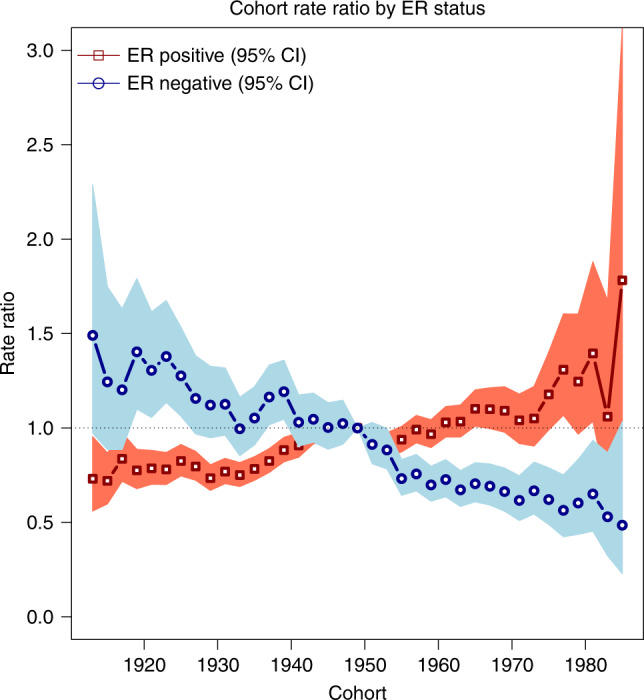
Birth cohort rate ratios (CRR) for breast cancer incidence rates in Scotland by ER status. CRR describes the incidence rates for each birth cohort relative to the 1949 birth cohort.

Compared with the women born in 1949, ER+ tumour incidence was higher among more recent birth cohorts. In contrast, ER− incidence was lower for more recent birth cohorts compared with the cohort born in 1949. CRRs compared with women born in 1949 ranged from 0.7 for women born in 1913 to 1.8 for women born in 1985 for ER+ tumours, and from 1.5 for women born in 1913 to 0.5 for women born in 1985 for ER− tumours (Fig. 3). The combination test for ER+ tumours revealed cohort effects beyond the log-linear trend shown by the net drift (*p* value < 0.0001), but the test for ER– tumours failed to reach significance (*p* value = 0.14).

## Discussion

This study demonstrates that, in Scotland, temporal trends of breast cancer incidence were distinct by molecular subtypes, with increases for ER+ and decreases for ER− tumours between 1997 and 2016. With respect to ER+ tumours, their incidence increased for all ages for the study period, but particularly among women of screening ages 50–69 years, with the largest increases occurring from around 1997 to 2011 followed by modest declines. In contrast, the incidence of ER− cancers decreased among all ages till the early 2000s. Finally, we noted cohort effects such that, in comparison with women born around 1950, women of older generations (those born in the 1910s–1940s) had a lower risk of ER+ tumours, whereas there was no significant evidence for cohort effects for ER− tumours. Further analysis of the incidence trends by subtype (as defined by ER/HER2 combinations) generally showed similar results to those observed by ER status only. ER+/HER2− (surrogate for luminal A) tumours followed the same pattern as all ER+ tumours. However, our findings suggest a significant increase in the rarer and more aggressive ER−/HER2− breast cancers among women 20–49 years of age, similar to recent increases noted in the United States that need careful future monitoring.^36^ Our data affirm that future incidence and survival reporting should be monitored by molecular subtypes to inform clinical planning and cancer control programmes.

Consistent with reports from the United States, Denmark and Ireland,^7–9^ our data show for the first time in a UK national cancer registry, contrasting temporal trends of breast cancer incidence by ER status, and suggest the presence of aetiologic heterogeneity with distinct patterns by period, age at diagnosis and birth cohort. Previous studies have shown estimated annual increases in the age-standardised rate of breast cancer from early 1990s to 2010 for ER+ ranging from 0.1 to 3% and declines for ER− ranging from −1.9 to −3.4%.^7–9^ The Scottish Cancer Registry’s detailed tumour hormone receptor data have been used to describe trends in incidence patterns of breast cancer. Specifically, it was previously reported that there were declines in ER+ tumours among women 50–64 years of age that were statistically significant by 2005.^12^ These findings were attributed to reduction in menopausal hormone (MH) use (also known as hormone-replacement therapy), which had been shown to be associated with increased risk of breast cancer. Unlike the previous analysis, we excluded women with a previous malignancy, imputed missing ER status and used individuals rather than tumours as the numerator for incidence rates, but the findings were similar for comparable years, confirming that MH resulted in more women diagnosed with breast cancer. The declines in breast cancer incidence coincident with decreased MH use observed in Scottish data have also been shown in the United States,^37^ Sweden, Norway^38^ and France.^39^ We observed consistent increases over time for ER+ tumour incidence beyond 2002, after which MH use declined. Based on recent reports on the association of MH use and breast cancer risk, MH has been estimated to contribute to 1 in 20 breast cancers diagnosed worldwide since 1990.^40^ In more recent years, when MH use has declined, MH has been estimated to have an approximate 5-year lag time to breast cancer incidence, and contribute to 2.3% of breast cancers in Scotland in recent years. Despite reductions in MH use from 2005 to 2011, the incidence of breast cancer continued to increase. In addition to the long-term effects of previous MH use, other factors, such as screening efficiency and obesity, are also likely to contribute to time trends in breast cancer incidence.

Mammographic screening is likely to be an important contributing factor to the increased incidence of ER+ tumours we observed from 1997 to 2011. In Scotland, the breast screening programme was established in 1988 with full national coverage attained in 1991.^41^ Scotland’s breast screening programme was introduced earlier than in other countries that have evaluated breast cancer incidence trends by ER status (i.e. 2000 in Ireland and 2010 in Denmark; in the United States, although there are no national screening programmes, in the Kaiser Permanente Health Management Organization, uptake of screening to 75% of eligible women was seen starting in 1993)^37^. From 1994 to 2003, women 50–64 years of age in Scotland were invited for screening, with extension in 2003 to include women aged 65–70 years. Over the course of the entire study period in Scotland, the mammographic screening programme had around 75% uptake. Our data showing that ER+ tumours are more likely to be screen-detected than ER− tumours (53% vs. 30%), and our APC model results showing incidence of ER+ tumours greatest for those of screening ages between 65 and 72 years, suggest that some of the increases observed in ER+ tumours are likely to be due to detection of prevalent disease in these older women. A similar pattern was also observed in the previous report.^12^ Our analysis among women of screening age showed that the trend for screen-detected ER+ breast cancers is similar to that of the overall ER+ breast cancer incidence seen in this age group, strongly suggesting that mammographic screening is better at detection of ER+ than ER− breast cancers. Detecting ER− breast cancers has remained a challenge—they tend to present at younger ages, as larger tumours, and have fewer targeted treatments unlike ER+ breast cancers.^42^ The natural history of breast cancer suggests a complicated aetiology when evaluating screen-detected tumours.^43^ Our data suggest that ER+ screen-detected tumours have been significantly increasing over the time period of our study although, in more recent years, the incidence has stabilised or perhaps declined slightly, which we intend to continue monitoring.

Yen and colleagues aimed to determine risk factors and molecular tumour markers that might be associated with screen-detected tumours using data on 1924 screen-detected and 1001 interval-detected cancer cases diagnosed in Sweden.^44^ They found that higher BMI, older age at first birth, higher breast density (the radiologic appearance of the breast) and family history of breast cancer were significant positive contributors to tumours that are diagnosed through mammography screening. These data are consistent with increasing obesity and advancing ages at first birth in the population contributing towards likely increasing risk and therefore incidence of ER+ tumours. Furthermore, specific molecular subtypes, such as ER− breast cancers and the subset of ER− basal-like tumours, were more likely to be interval cancers. Predictive modelling of breast cancer has been proposed as a potential tool for personalised medicine and risk-stratified screening,^45–47^ and future efforts might be used within screening programmes to improve the detection of more aggressive ER− breast cancers, particularly amongst those at higher risk of developing such cancers. With increased technologic advances in imaging modalities, it will be important to assess how these impact screen-detected tumours, and whether they can also improve detection for more aggressive ER-negative tumours that are more likely to be diagnosed outside of most screening programmes’ age ranges. With increasing emphasis on efficiency in maximising limited resources, modelling studies on stratified screening using UK data suggest that such approaches could improve the cost-effectiveness of the screening programme, reduce overdiagnosis and maintain the benefits of screening.^48^

The strengths of our study are the high quality of the longitudinal data collected within the Scottish cancer registry, the first one in the United Kingdom that routinely started recording molecular marker data (ER status from 1997 and PR and HER2 status from 2009). Marker data can be used to monitor and describe incidence trends in the future and for other types of cancer that display heterogeneity. Further, monitoring breast cancer incidence by molecular subtypes can help the NHS allocate resources for treatment and prevention, and lead to the identification of high-risk groups of women for which to implement future prevention programmes and treatments.

A potential limitation of our study is imputation of ER status for 8% of the population and the assumptions used, which were that ER/HER2 data have the same chance of being missing among each cohort of patients by year and age at diagnosis. For this assumption to be wrong, there would have to be a confounder associated with ER status that would influence whether ER status was tested and recorded. This scenario seems unlikely in Scotland’s health service where guidelines are used to inform investigation and treatment. Missingness is more likely to reflect administrative omissions, and geographic uptake in reporting ER status. This assumption has been used in US, Denmark and Irish data.^7–9^ Performing multiple imputation using additional individual-level covariates would be more important when describing survival. An extended imputation model for individuals that incorporated the American Joint Committee on Cancer TNM stage^49^ and tumour grade in addition to age and year of diagnosis found that the overall imputed counts were very similar to those obtained using the simpler model that contained just age and year of diagnosis.^15^ Therefore, redistributing the relatively small percentage of missing receptor status in cases within each single year of age at diagnosis and calendar year of diagnosis according to the distribution observed for that specific cohort of patients is appropriate for estimating incidence trends.

Another limitation of our study is the absence of individual-level risk factor data, including participation in breast screening programmes in prior years to define interval breast cancers and stage data. However, in future studies, it should be possible to identify some key factors using linked data including detailed cohort data. The United Kingdom is renowned for its high-quality, longitudinal data and the ability to perform linkage studies using a unique identifier. Hence, we envision future analysis using the cancer registry linked to other datasets, including community prescription drug records, mammography imaging, maternity and hospital records to provide more detailed information on the role and patterns of key risk factors in breast cancer incidence trends. Another limitation of the study is the lack of mRNA expression assays for the classification of the molecular subtypes of breast cancer. In our study, markers measured by IHC are used as surrogates for the molecular subtypes, which are reasonably good proxies, but mRNA profiling data would be considered a gold standard for intrinsic-subtype classification.^13^

In conclusion, incidence trends of breast cancer in Scotland differ by ER status, and are consistent with trends observed in other countries. It will be important to monitor whether ER+ tumour incidence stabilises or reduces over time. Additional data are needed to establish whether incidence of HER2+ tumours, which are ER−, remains low since their treatment involves monoclonal antibodies, such as trastuzumab and pertuzumab,^13,50^ which are amongst the more expensive breast cancer treatments used by the NHS. Further research should be focused on monitoring incidence trends by subtype because of the marked risk, detection and treatment differences for breast cancer subtypes.

## Supplementary information

Supplemental material_revision

## Data Availability

The data used in this study can be accessed through application to electronic Data Research and Innovation Service (eDRIS), a part of the Information Services Division of NHS Scotland.
