# Supplementary material for: Distinct temporal trends in breast cancer incidence from 1997 to 2016 by molecular subtypes: a population-based study of Scottish cancer registry data
Source: Br J Cancer. 2020 Jun 19;123(5):852–9. doi: 10.1038/s41416-020-0938-z (PMC7463252; doi:10.1038/s41416-020-0938-z)
Supplement: Supplementary file 1 — Supplemental material_revision [file 41416_2020_938_MOESM1_ESM.docx]

**Supplemental Material**

**Supplemental Figure 1: Flowchart of selection of study population for analysis**

Number of people

n=74,324

Men

n=398

Tumours s in women

n= 91,185

Number of women

n= 73,926

Women with other primary malignancy prior to invasive breast cancer diagnosis

n=1,709

Number of women

n= 72,217

Women with single invasive tumour

n=68,564

Women with multiple invasive breast tumours

n=3,653

We excluded cases 1) diagnosed in males; 2) tumours in women with a previous primary malignant cancer in a different organ and; 3) women with tumours of unknown behaviour, as their aetiology may be different or uncertain in comparison to the aetiology of primary invasive breast cancers.

**Supplemental Figure 2: Age-standardised breast cancer incidence rates by ER and HER2 status in Scotland for 1997-2016**


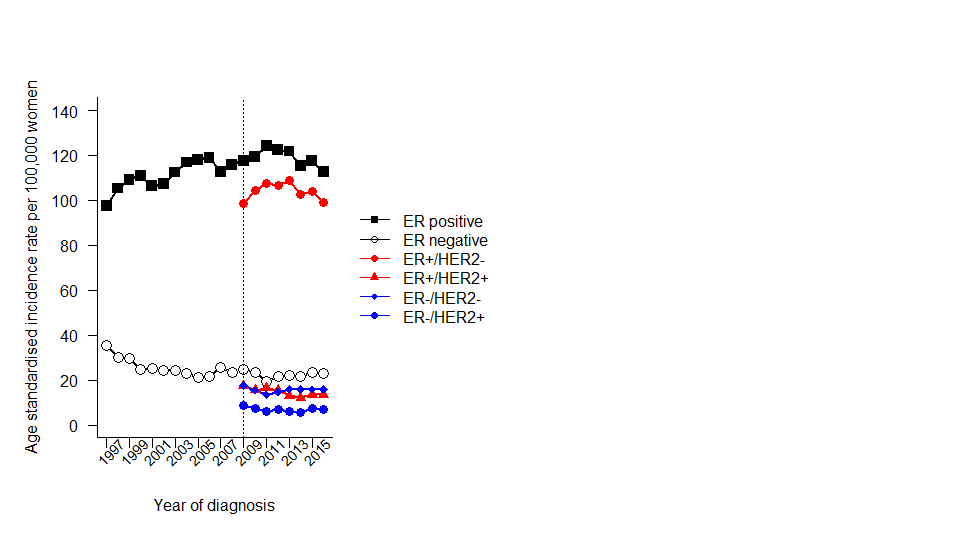


ER/HER2 combinations were available from 2009 to 2016, N=31,099. Dotted line at year 2009 denotes when HER2 status started to be collected in the Scottish cancer registry.

**Supplemental Figure 3: Incidence rates of Luminal B-like, HER2-enriched-like and triple negative tumours by age group from 2009-2016, imputed for missing ER and HER2 status**


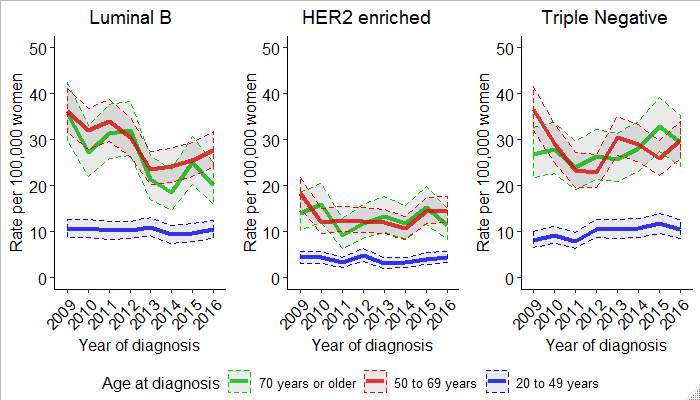


**Supplemental Figure 4: Age Period Cohort model results for age-specific incidence trends by ER+ (panel a) and ER- (panel b) breast cancers in Scotland**

| a)  **ER positive**  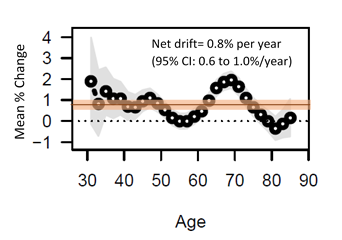 |
| --- |
| b)  **ER negative**  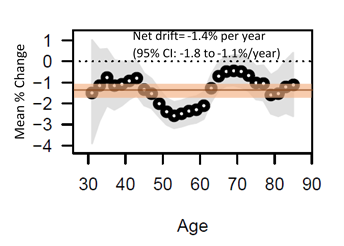 |

The global net drift (orange line) is analogous to the estimated annual percentage change (EAPC) in the age‐standardized incidence rate; whereas the local net drifts provide estimates of the corresponding EAPCs for individual age groups. Shaded area surrounding net drift line indicates 95% CI of the estimate. Dotted line in black indicates a mean % change of zero (no change in incidence).

**Supplemental Table 1: Joinpoint regression results of breast cancer incidence by ER/HER2 status by age groups from 2009-2016**

| ER/HER2 Status | Age groups | Rate in 2009 | Rate in 2016 | Change in rate from 2009 to 2016 N per 100,000 (%) | Average Annual Percentage Change | Years before joinpoint | EAPC for period before joinpoint with 95%CI | Years after joinpoint | EAPC for period after joinpoint with 95% CI |
| --- | --- | --- | --- | --- | --- | --- | --- | --- | --- |
| ER+/HER2- (Luminal A like) | All ages | 98.6 | 99.1 | 0.5 (0.5%) | 0.3% (-1.7, 2.3) | 2009-2011 | 5% (-5.1, 16.1) | 2011-2016 | -1.6% (-3.7, 0.6) |
|  | 20-49 years | 43.7 | 41.7 | -2 (-4.6%) | 0.2% (-1.5,1.9) | **No significant change point identified from 2009-2016** | | | |
|  | 50-69 years | 201.3 | 210.4 | 9.1 (4.5%) | 0.3% (-1.8, 2.6) | 2009-2011 | 6.9% (-4.2, 19.2) | 2011-2016 | -2.1% (-4.4, 0.1) |
|  | 70+ years | 221.4 | 214.7 | -6.7 (-3%) | -0.2% (-1.7, 1.4) | **No significant change point identified from 2009-2016** | | | |
| ER+/HER2+ (Luminal B like) | All ages | 17.4 | 13.6 | -3.8 (-22%) | **-4%(-6.6, -1.4)** | **No significant change point identified from 2009-2016** | | | |
|  | 20-49 years | 10.3 | 10.3 | 0 | -0.4% (-2.4, 1.5) | **No significant change point identified from 2009-2016** | | | |
|  | 50-69 years | 33.6 | 27.0 | -6.6 (-20%) | **-4.4% (-7.7, -0.9)** | **No significant change point identified from 2009-2016** | | | |
|  | 70+ years | 35.0 | 19.8 | -15.2 (-43%) | **-7.2% (-12.1, -1.9)** | **No significant change point identified from 2009-2016** | | | |
| ER-/HER2+ (HER2 enriched like) | All ages | 9.0 | 7.1 | -1.9 (-21%) | -2.4% (-7.3, 2.8) | **No significant change point identified from 2009-2016** | | | |
|  | 20-49 years | 4.5 | 4.5 | 0 | -1.9% (-8.6, 5.4) | **No significant change point identified from 2009-2016** | | | |
|  | 50-69 years | 20.6 | 15.1 | -5.5 (-27%) | -3.1% (-9.6, 3.9) | **No significant change point identified from 2009-2016** | | | |
|  | 70+ years | 14.7 | 11.5 | -3.2 (-22%) | -2% (-8.6, 5.1) | **No significant change point identified from 2009-2016** | | | |
| ER-/HER2- (Triple negative) | All ages | 17.5 | 16.1 | -1.4 (-8%) | -0.7% (-3.2, 1.8) | 2009-2011 | -9.8% (-20.4, 2.3) | 2011-2016 | **3.2% (0.3, 6.1)** |
|  | 20-49 years | 8.6 | 10.8 | 2.2 (26%) | **4% (0.9, 7.2)** | **No significant change point identified from 2009-2016** | | | |
|  | 50-69 years | 40.2 | 30.5 | -9.7 (-24%) | -3.7% (-12.5, 6.0) | 2009-2011 | -18.6% (-49.0, 30.1) | 2011-2016 | 3% (-7.9, 15.2) |
|  | 70+ years | 29.0 | 30.1 | 1.1 (3.8%) | 1.5% (-0.9, 3.9) | **No significant change point identified from 2009-2016** | | | |

Bold estimates are significantly different from 0 (p<0.05). EAPC estimated annual percentage change. AAPC estimated average annual percent change. Joinpoint regression was performed using the estimated counts corrected for missing ER/HER2 status, and analysis corrects for multiple testing using Bonferroni correction (See methods section)
